# Supplementary material for: Splice-Junction-Based Mapping of Alternative Isoforms in the Human Proteome
Source: Cell Rep. Author manuscript; Available in PMC 2020 Jan 15. (PMC6961840; doi:10.1016/j.celrep.2019.11.026)
Supplement: 2 [file NIHMS1546469-supplement-2.zip › DF1/PXD006675/VentricularSeptum/VentricularSeptum_12_TTN_SVPEPPPPKPVEEVEVPTVTK.pdf]

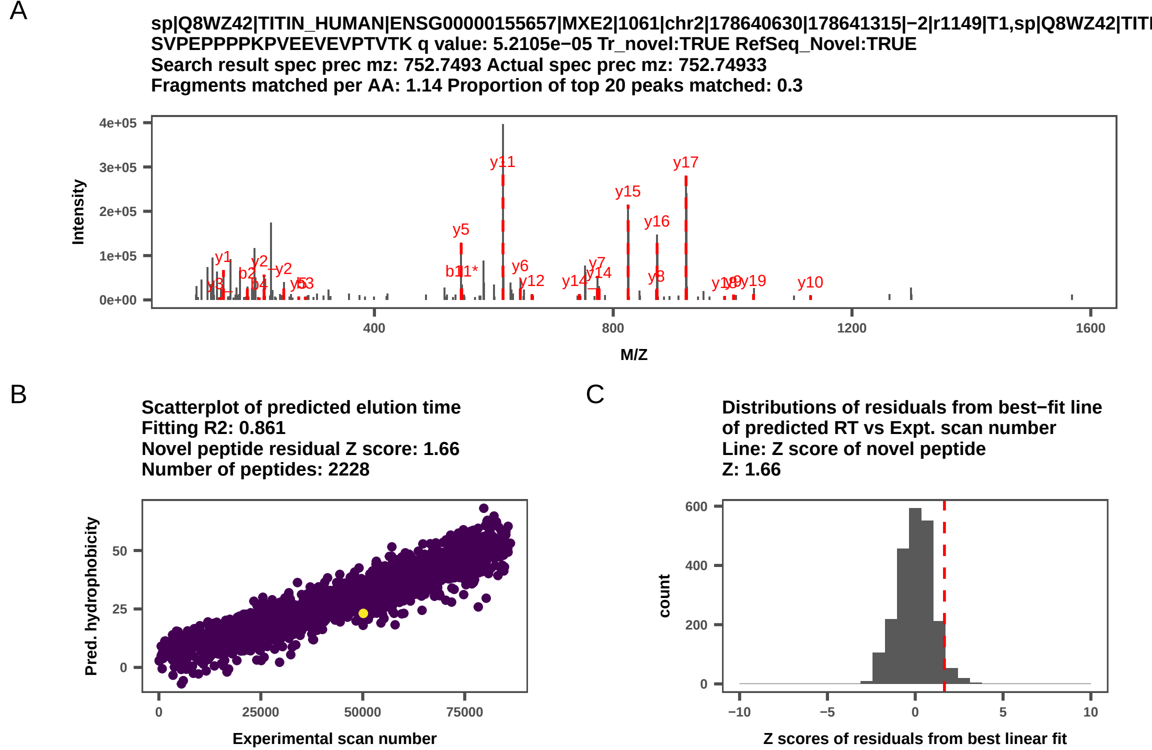
A
sp|Q8WZ42|TITIN_HUMAN|ENSG00000155657|MXE2|1061|chr2|178640630|178641315|−2|r1149|T1,sp|Q8WZ42|TITI
SVPEPPPPKPVEEVEVPTVTK q value: 5.2105e−05 Tr_novel:TRUE RefSeq_Novel:TRUE
Search result spec prec mz: 752.7493 Actual spec prec mz: 752.74933
Fragments matched per AA: 1.14 Proportion of top 20 peaks matched: 0.3
Intensity
4e+05
3e+05
2e+05
1e+05
0e+00
y11
y17
y15
y16
y5
y1
y2
b2
y2
y6
y12
y7
y14
y14
y8
y19
y10
400
800
1200
1600
M/Z
B
Scatterplot of predicted elution time
Fitting R2: 0.861
Novel peptide residual Z score: 1.66
Number of peptides: 2228
Pred. hydrophobicity
50
25
0
25000
50000
75000
Experimental scan number
C
Distributions of residuals from best−fit line
of predicted RT vs Expt. scan number
Line: Z score of novel peptide
Z: 1.66
count
600
400
200
0
−10
−5
5
10
Z scores of residuals from best linear fit
